# Early lenalidomide treatment for low and intermediate‐1 International Prognostic Scoring System risk myelodysplastic syndromes with del(5q) before transfusion dependence

**DOI:** 10.1002/cam4.523

**Published:** 2015-09-17

**Authors:** Esther N. Oliva, Michael Lauseker, Maria Antonietta Aloe Spiriti, Antonella Poloni, Agostino Cortelezzi, Giuseppe A. Palumbo, Enrico Balleari, Grazia Sanpaolo, Antonio Volpe, Alessandra Ricco, Francesca Ronco, Caterina Alati, Maria Grazia D'Errigo, Irene Santacaterina, Andrea Kündgen, Ulrich Germing, Roberto Latagliata

**Affiliations:** ^1^ Hematology UnitBianchi‐Melacrino‐Morelli HospitalReggio CalabriaItaly; ^2^ Institute for Medical Information Science, Biometry and EpidemiologyLudwig‐Maximilians‐UniversityMunichGermany; ^3^ Department of Clinical and Molecular MedicineLa Sapienza UniversityRomeItaly; ^4^ Marche PolytechnicAnconaItaly; ^5^ IRCCS Ca' Granda FoundationMaggiore General HospitalUniversity of MilanMilanItaly; ^6^ Vittorio Emanuele General HospitalUniversity of CataniaCataniaItaly; ^7^ Hematology UnitSan Martino HospitalGenoaItaly; ^8^ Hematology DivisionCasa Sollievo della Sofferenza HospitalSan Giovanni RotondoItaly; ^9^ San G Moscati HospitalAvellinoItaly; ^10^ University of BariBariItaly; ^11^ Medical Genetic UnitBianchi‐Melacrino‐Morelli HospitalReggio CalabriaItaly; ^12^ Department of Hematology, Oncology and Clinical ImmunologyHeinrich‐Heine‐University of DüsseldorfDüsseldorfGermany; ^13^ Department of Cellular Biotechnology and HematologyLa Sapienza UniversityRomeItaly

**Keywords:** 5q deletion syndrome, anemia, lenalidomide, myelodysplastic syndromes, quality of life

## Abstract

Lenalidomide is approved for the treatment of transfusion‐dependent (TD) del(5q) myelodysplastic syndromes (MDS). However, few data are available in patients with transfusion‐independent (TI) del(5q) MDS. In the first, observational, part of this 2‐part study, we assessed the impact of transfusion dependence on overall survival (OS) and non‐leukemic death in untreated del(5q) MDS patients who were TD (*n* = 136), TI with hemoglobin (Hb) ≥10 mg/dL (*n* = 88), or TI with Hb <10 mg/dL (*n* = 96). In the second, interventional, part we assessed the quality‐of‐life (QoL) benefits and clinical efficacy of lenalidomide (10 mg/day) in 12 patients with TI del(5q) MDS and Hb <10 mg/dL. In the untreated population, OS was significantly longer in TI than in TD patients (TI [Hb ≥10 g/dL], 108 months; TI [Hb <10 g/dL], 77 months; TD, 44 months). Transfusion dependence also negatively impacted non‐leukemic death rates. In the interventional part of the study, baseline Hb levels were found to correlate significantly with physical (*R* = 0.666, *P* = 0.035) and fatigue (*R *=* *0.604, *P = *0.049) QoL scores. Median physical QoL scores improved significantly after 12 weeks' treatment with lenalidomide (+12.5; *P *=* *0.020). Evaluable TI patients experienced early increases in Hb levels, and all attained an erythroid response. Our findings suggest that TI patients with moderate anemia may benefit from early treatment with lenalidomide.

## Introduction

Lenalidomide is approved in several countries for the treatment of transfusion‐dependent (TD) patients with anemia related to International Prognostic Scoring System (IPSS)‐defined low‐ or intermediate‐1 (Int‐1)‐risk myelodysplastic syndromes (MDS) and an isolated deletion of the long arm of chromosome 5 [del(5q)]. Treatment induces erythroid and cytogenetic responses in a high proportion of patients 1, 2, 3, and erythroid responses appear to be associated with reduced risk of disease progression, improved survival, and quality‐of‐life (QoL) benefits 1, 4.

More than half of patients with del(5q) MDS are transfusion independent (TI) at the time of diagnosis 5, 6; however, most will ultimately become TD during the course of their disease 7. Erythropoiesis‐stimulating agents (ESAs) are widely used off label to treat anemia associated with MDS, with response rates ranging from a maximum of 68% to a minimum of 19% 8. A meta‐analysis of 30 studies published between 1990 and 2006, which included 1314 MDS patients, identified the following as being predictive of response to ESA treatment: low transfusion dependence; a morphological diagnosis of refractory anemia (RA)/RA with ringed sideroblasts; treatment with a fixed‐dose versus a weight‐based ESA regimen; shorter interval between diagnosis and treatment initiation; and lower baseline serum erythropoietin level 8. However, response rates to ESAs are lower, and the duration of response is shorter, in patients with MDS with del(5q) than in MDS patients without this aberration (39% vs. 52%, *P *=* *0.10, and 13 months vs. 27 months, *P *=* *0.003, respectively, both according to International Working Group 2006 criteria) 9.

While transfusions may help to alleviate the occurrence and symptoms of anemia, prolonged transfusion dependence is associated with reduced survival in patients with MDS 10, 11, 12, 13, including those with del(5q) 6. For example, in a report of 381 untreated patients with low‐ or Int‐1‐IPSS risk del(5q) MDS, median survival among TD patients was 44 months, compared with 97 months in TI patients (*P *<* *0.0001) 6. Transfusion dependence is also associated with increased incidence and severity of comorbidities in patients with MDS 5, 11. Of particular concern are cardiac events, as cardiac failure has been identified as the main cause of non‐leukemic death in this population 5. Furthermore, the risk of cardiac death is significantly increased in patients with chronic anemia 14, and a hemoglobin (Hb) level of about 10 g/dL has been suggested as the threshold for cardiac remodeling 15. Consequently, active treatment options with the potential to prevent/delay transfusion dependence and improve QoL would be advantageous for patients with del(5q) MDS.

To date, patients with TI del(5q) MDS have typically been excluded from clinical trials with lenalidomide, partly as a result of concerns that lenalidomide may accelerate progression to acute myeloid leukemia (AML) 16, 17, 18. However, the most comprehensive analysis available to date suggests that these concerns are unfounded 18. Therefore, in view of the detrimental effects of prolonged transfusion dependence, we investigated the rationale for early intervention with lenalidomide in TI patients with anemia (defined as Hb <10 g/dL) and low or Int‐1 IPSS risk del(5q) MDS. We carried out a 2‐step, retrospective analysis to: (i) assess the influence of transfusion dependence and Hb levels on overall survival (OS) and non‐leukemic death in an untreated cohort of patients with del(5q) MDS (observational study)^6^ and (ii) evaluate the efficacy and QoL benefits of lenalidomide in a subset of Italian patients with TI del(5q) MDS enrolled in the phase 2 RevMDS trial (interventional study) 4.

## Subjects and Methods

### Observational part of study

The cohort of 381 patients has been described previously 6. In brief, the cohort comprised patients with a documented diagnosis (and an exact date of diagnosis) by bone marrow examination and cytogenetic assessment of low or Int‐1 IPSS risk del(5q) MDS from nine centers and registries in Europe, the USA, and Australia. All patients were untreated and receiving best supportive care (defined as transfusions, chelation therapy, or ESAs). A total of 71 patients who had received lenalidomide were initially included, but were censored at the start of treatment.

Transfusion dependence was defined as a requirement for at least 4 units of red blood cell transfusions within 8 weeks 6. Patients were divided into three groups according to transfusion status: TD, TI with baseline Hb values of ≥10 g/dL, and TI with baseline Hb values of <10 g/dL. 10 g/dL was established as the cut‐off Hb level to confirm the impact of chronic anemia on survival. OS in each group was calculated by Kaplan–Meier analysis. For the event of non‐leukemic death, cumulative incidences were estimated, considering progression to AML as a competing event. For both analyses, patients with no observed events were censored at the last follow‐up. Hazard ratios (HRs) for death (and non‐leukemic death) were calculated using Cox proportional hazards models for TI patients (with Hb ≥ or <10 g/dL) versus TD patients.

### Interventional part of study

The lenalidomide cohort comprised patients from the phase 2, multicenter, non‐randomized, single‐arm, open‐label RevMDS lenalidomide trial (registered: EudraCT No. 2008‐000547‐34) 4. This trial enrolled a total of 45 patients with anemia and low or int‐1 IPSS risk del(5q) MDS with or without additional cytogenetic abnormalities, irrespective of transfusion status. The group comprised 31 TD patients and 14 TI patients (based on current MDS criteria). The focus of the analysis presented here was the 12 TI patients with Hb <10 g/dL.

All patients received oral lenalidomide at a starting dose of 10 mg once daily for up to 12 months, or until unacceptable toxicity, lack of response, disease progression, or relapse following erythroid improvement. The primary endpoint of the study was QoL. Secondary endpoints were efficacy and survival.

QoL during treatment was assessed using the MDS‐specific, health‐related QoL instrument QOL‐E© version 2 19, 20, which was completed at baseline and at 8, 12, 24, and 52 weeks' follow‐up. The QOL‐E© questionnaire comprises two items concerning general perceptions of well‐being, and 26 items addressing physical, functional, social, sexual, fatigue, and disease‐specific domains. Higher scores in each domain (range, 0–100) reflect better QoL. Missing items were not substituted to score domains. This analysis focuses on changes from baseline to 12 weeks.

Hb was measured as part of a complete blood count performed weekly for the first 8 weeks, and every 15 days thereafter. Cytogenetic studies were carried out at a central laboratory at baseline and every 12 weeks during the study period.

### Statistical analysis

Descriptive statistics were calculated, and parametric and non‐parametric tests were applied. The prespecified criterion for significance was *P *<* *0.05. Means and standard deviations were used to describe normally distributed data. Non‐normally distributed data were summarized using medians and interquartile ranges (IQRs) or frequencies (percentage), as appropriate. Between‐group and within‐patient comparisons were performed using paired *t*‐tests and the Wilcoxon test respectively. Spearman's rank correlation coefficient was used to investigate associations between pairs of continuous variables. Univariate Kaplan–Meier analysis was used to evaluate survival in the different subgroups.

## Results

### Observational part of study

This analysis was based on 320 patients, including 136 TD patients, 88 TI patients with Hb ≥10 g/dL, and 96 TI patients with Hb <10 g/dL. In total, 61 patients were excluded due to unknown transfusion status (*n* = 54) and missing baseline Hb data (all TI patients; *n* = 7). Baseline characteristics are shown in Table 1. Groups were well‐matched in terms of age and gender distribution, and in the prevalence of neutropenia and thrombocytopenia. It is worthy of note, however, that RA with excess blasts‐1 (RAEB‐1) was significantly more prevalent among TD than among TI patients, regardless of Hb level (*P *=* *0.030). RAEB‐1 rates did not differ significantly between the two TI subgroups (*P *=* *0.555).

**Table 1:** Observational part of study: baseline patient characteristics

Characteristic	TD, *n* = 136	TI with Hb ≥10 g/dL, *n* = 88	TI with Hb <10 g/dL, *n* = 96
Age: median (range), years	66 (28–91)	67 (33–95)	66.5 (31–91)
Gender, *n* (%)			
Male	44 (32)	24 (27)	26 (27)
Female	92 (68)	64 (73)	70 (73)
IPSS status, *n* (%)			
Low	50 (40)	56 (67)	40 (46)
Int‐1	76 (60)	28 (33)	47 (54)
Neutropenia, *n* (%)	48 (38)	29 (35)	35 (40)
Thrombocytopenia, *n* (%)	19 (14)	12 (14)	5 (5)
WHO classification, *n* (%)			
RA	11 (8)	7 (8)	7 (7)
RARS	0	5 (6)	3 (3)
RCMD	18 (13)	11 (13)	7 (7)
5q syndrome	87 (64)	58 (66)	74 (77)
RAEB‐1	20 (15)	7 (8)	5 (5)
Karyotype 21, *n* (%)			
Low risk^a^	113 (83)	67 (76)	79 (82)
Intermediate risk^b^	18 (13)	17 (19)	13 (14)
High risk^c^	5 (4)	4 (5)	4 (4)

Hb, hemoglobin; Int‐1, intermediate‐1; IPSS, International Prognosis Scoring System; RA, refractory anemia; RAEB‐1, RA with excess blasts‐1; RARS, RA with ringed sideroblasts; RCMD, refractory cytopenia with multilineage dysplasia; TD, transfusion‐dependent; TI, transfusion‐independent; WHO, World Health Organization.

a Isolated del(5q).

b All karyotoypes not defined as low‐ or high‐risk karyotypes.

c del(5q) plus either chromosome 7 abnormality or ≥2 additional aberrations.

**Figure 1:**
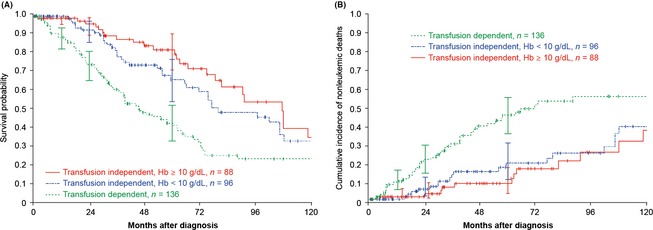
Observational part of study: (A) overall survival and (B) cumulative incidence of non‐leukemic deaths by transfusion dependence and hemoglobin levels at diagnosis.

OS and cumulative incidence of non‐leukemic death at 2 and 5 years' follow‐up are shown in Table 2. OS was significantly longer for both groups of TI patients than for TD patients (Fig. 1A), with the following medians: 108 months in TI (Hb ≥10 g/dL) patients, 77 months in TI (Hb <10 g/dL) patients, and 44 months in TD patients. HRs were 0.44 (95% confidence interval [CI], 0.29‐0.68, *P *<* *0.001) for TI (Hb ≥10 g/dL) versus TD, and 0.55 (95% CI, 0.38–0.81, *P *=* *0.002) for TI (Hb <10 g/dL) versus TD. In the TI subgroup, OS did not differ significantly between patients with Hb ≥10 g/dL and those with Hb <10 g/dL (HR 0.80; 95% CI, 0.50–1.29; *P *=* *0.355). In terms of non‐leukemic death, survival was again significantly longer for both TI groups than for TD patients (Fig. 1B). HRs were 0.34 (95% CI, 0.19–0.61, *P *<* *.001) for TI (Hb ≥10 g/dL) versus TD, and 0.42 (95% CI, 0.25–0.71, *P *=* *.001) for TI (Hb <10 g/dL) versus TD. The risk of non‐leukemic death was similar in the two TI patient groups (HR 0.81; 95% CI, 0.42–1.57; *P *=* *0.533).

**Table 2:** Observational part of study: overall survival probability and cumulative incidence of non‐leukemic deaths by transfusion dependence and hemoglobin levels at diagnosis

	TD, *n* = 136	TI with Hb ≥10 g/dL, *n* = 88	TI with Hb <10 g/dL, *n* = 96
2‐year survival probability (95% CI), %	73.2 (64.8–80.9)	95.8 (89.9–99.2)	92.5 (85.8–97.2)
5‐year survival probability (95% CI), %	41.3 (31.6–51.2)	81.6 (70.7–90.5)	65.4 (53.4‐76.4)
Survival: median (95% CI), months	44 (37–61)	108 (82–129)	77 (66–109)
2‐year cumulative incidence of non‐leukemic deaths (95% CI), %	21.3 (14.2–29.4)	2.8 (0.5–8.8)	5.2 (1.7–11.8)
5‐year cumulative incidence of non‐leukemic deaths (95% CI), %	46.0 (35.7–55.5)	8.3 (3.0–17.0)	19.7 (10.6–30.8)

CI, confidence interval; Hb, hemoglobin; TD, transfusion‐dependent; TI, transfusion‐independent.

### Interventional part of study

The baseline characteristics of the 45 patients enrolled in the phase 2 RevMDS lenalidomide trial have been reported previously 4; those for the 12 TI patients with Hb <10 g/dL are shown in Table 3. Among these patients, all of whom were female, median age was 68 years, and the majority were in the IPSS low‐risk category.

**Table 3:** Interventional part of study: baseline characteristics of TI patients with hemoglobin <10 g/dL

	TI with Hb <10 g/dL, *n* = 12
Age: mean (IQR), years	68 (64–74)
Female, *n* (%)	12 (100)
Disease duration: median (IQR), years	1.7 (0.6–2.7)
IPSS risk category, *n* (%)	
Low	10 (83)
Int‐1	2 (17)
Karyotype, *n* (%)	
Isolated del(5q)	9 (75)
del(5q) + 1 additional abnormality^a^	3 (25)
WHO classification, *n* (%)	
RA	2 (16.7)
MDS with del(5q)	9 (75.0)
RCMD	1 (8.3)
Hemoglobin level: median (IQR), g/dL	9.0 (7.7–9.5)
Platelet count: median (range), 100 × 10^9^/L	3.6 (2.3–4.8)
ANC: median (range), 1 × 10^9^/L	2.2 (1.4–3.6)
Charlson comorbidity index score	
0	5 (42)
1	7 (58)

ANC, absolute neutrophil count; Int‐1, intermediate‐1; IPSS, International Prognosis Scoring System; IQR, inter‐quartile range; MDS, myelodysplastic syndromes; RA, refractory anemia; RCMD, refractory cytopenia with multilineage dysplasia; TI, transfusion‐independent; WHO, World Health Organization.

a With the exception of chromosome 7 abnormality.

On treatment with lenalidomide, TI patients experienced earlier improvements in Hb levels than TD patients. At 12 and 24 weeks, Hb changes were significantly greater in TI than in TD patients (3.6 ± 1.6 vs. 1.9 ± 2.1 g/dL, *P *=* *0.01, and 4.5 ± 1.6 vs. 3.1 ± 2.2 g/dL, *P *=* *0.04, respectively), but the difference was no longer significant at 36 or 52 weeks (4.2 ± 1.9 vs. 3.1 ± 2.1 g/dL, *P *=* *0.16, and 4.3 ± 2.3 vs. 3.5 ± 2.4 g/dL, *P *=* *0.35 respectively). However, the frequency of cytogenetic responses was not significantly greater in TD than in TI patients (68% vs. 58%, respectively, *P *=* *0.606). One of the TI patients refused to continue the study because of drug‐related myelosuppression, and was removed from subsequent analyses. All other patients were erythroid responders (defined as Hb increase ≥1.5 g/dL) 22.

Deaths occurred among TD patients only. There was no difference between the two groups in either disease progression (TD vs. TI: HR, 1.26; 95% CI, 0.32–4.88, *P *=* *0.75), or ‘death or progression’ (HR, 2.85; 95% CI, 0.83–9.81, *P *=* *0.10) 4.

### QoL changes in TI patients during treatment with lenalidomide

Individual QoL scores for the 12 TI patients at baseline and at week 12 are shown in Table 4. At baseline, nine patients reported poor QoL (score <60) in at least 1 domain. Baseline Hb levels were correlated with QOL‐E© physical (*R *=* *0.666, *P *=* *0.035) and fatigue scores (*R *=* *0.604, *P *=* *0.049). Overall, QoL scores improved within 8 weeks, particularly in the physical (baseline median, 43.8; IQR, 25.00–62.50; 8‐week median, 62.5; IQR, 46.88–78.13, *P *=* *0.063) and fatigue (baseline median, 71.4; IQR, 66.67–80.95; 8‐week median, 81.0; IQR, 76.19–86.90, *P *=* *0.062) domains.

**Table 4:** Interventional part of study: changes in hemoglobin levels and quality of life scores between baseline and week 12 in individual patients with transfusion‐independent MDS and hemoglobin <10 g/dL at baseline

Case	Hb, g/dL		QOL‐E© physical		QOL‐E© functional		QOL‐E© social		QOL‐E© fatigue		QOL‐E© MDS specific	
	Baseline	Week 12	Baseline	Week 12	Baseline	Week 12	Baseline	Week 12	Baseline	Week 12	Baseline	Week 12
1	6.5	12.8	37.50	62.50	NA	NA	50.00	NA	57.14	80.95	76.19	88.10
2	7.5	14.8	25.00	50.00	NA	NA	50.00	100	76.19	NA	NA	NA
3	7.6	12.3	37.50	62.50	22.22	55.56	50.00	25.00	71.43	57.14	40.48	23.81
4	7.8	11.0	12.50	50.00	22.22	NA	NA	NA	52.38	71.43	28.57	35.71
5	8.5	11.8	62.50	62.50	33.33	33.33	25.00	25.00	80.95	80.95	23.81	30.95
6	8.9	NA	25.00	NA	33.33	NA	0	NA	66.67	NA	92.86	NA
7	9.0	12.0	NA	62.50	33.33	33.33	62.50	87.50	80.95	80.95	73.81	80.95
8	9.1	13.6	50.00	75.00	11.11	33.33	0	0	66.67	76.19	52.38	38.10
9	9.3	11.0	NA	87.50	100	100	100	NA	66.67	76.19	83.33	NA
10	9.6	12.6	62.50	87.50	100	100	100	100	85.71	85.71	NA	92.86
11	9.7	12.2	62.50	62.50	22.22	22.22	NA	NA	NA	80.95	NA	NA
12	9.7	12.7	87.50	87.50	100	100	87.50	100	95.24	80.95	78.57	78.57

Hb, hemoglobin; MDS, myelodysplastic syndromes; NA, not available (value missing); QOL‐E©, Quality of Life E version 2, an MDS‐specific health‐related quality of life instrument 16, 17.

**Figure 2:**
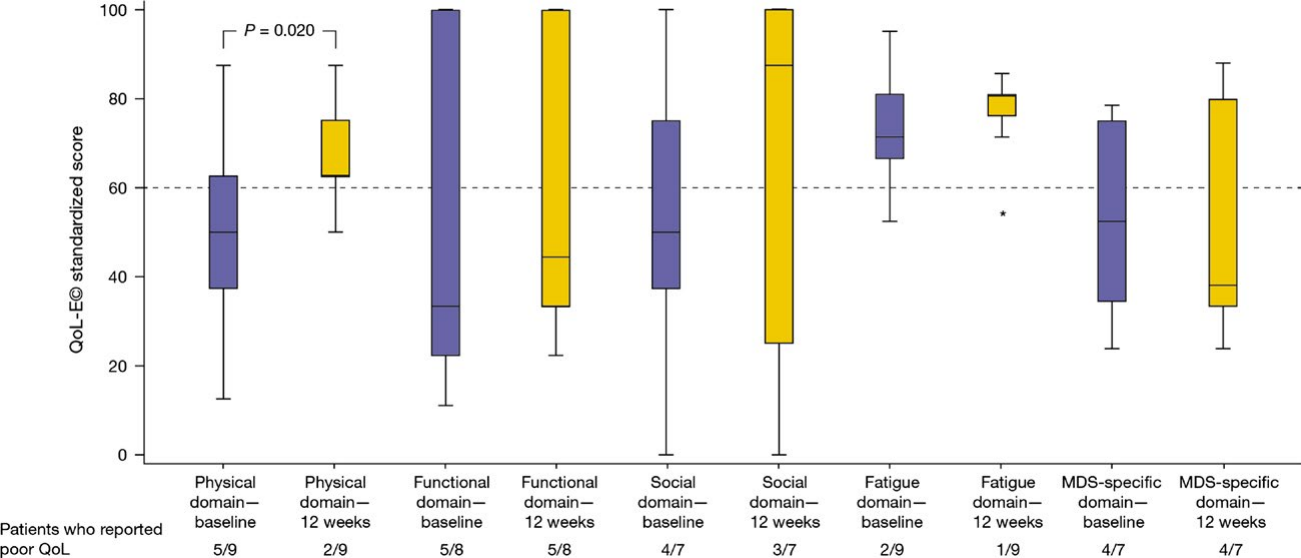
Interventional part of study: box plot showing descriptive statistics for quality of life changes from baseline to 12 weeks in transfusion‐independent patients during lenalidomide treatment. *indicates outlying data point. MDS, myelodysplastic syndromes.

**Figure 3:**
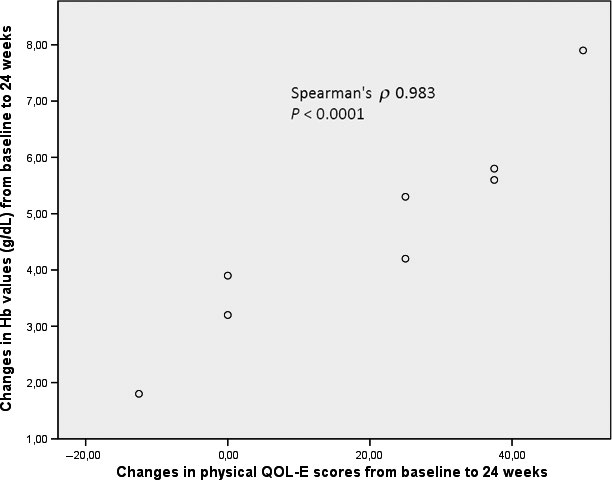
Interventional part of study: changes of physical quality of life‐E scores (*x*‐axis) from baseline to 24 weeks significantly correlate with changes in hemoglobin levels (*y*‐axis).

Changes at 12 weeks were significant for physical QoL scores (baseline median, 50.0; IQR, 31.25–62.50; 12‐week median, 62.5; IQR, 56.25–81.25, *P *=* *0.020; Fig. 2); improvements were observed in five of six patients with poor baseline physical QoL. Baseline Hb levels in these patients ranged from 6.5 to 9.1 g/dL. The variation in physical scores from baseline to 24 weeks were significantly correlated with changes in Hb levels (Fig. 3). No other significant differences were observed; however, the proportion of patients who reported poor QoL decreased, and median scores increased in the functional, social, and fatigue domains after 12 weeks. Interestingly, in the MDS‐specific domain, the mean MDS‐specific score was slightly lower at 12 weeks than at baseline.

## Discussion

The first, observational, part of this study assessed OS and the cumulative incidence of non‐leukemic death according to transfusion dependence and Hb levels in patients with del(5q) MDS managed by best supportive care only. This analysis builds on that previously reported by Germing et al. 6, who, using a multivariate analysis in the same cohort, found that transfusion dependence, anemia, thrombocytopenia, cytogenetic status, and age were all significant predictors of survival. Our subgroup analysis revealed that both OS and non‐leukemic death rates were significantly improved in TI patients, regardless of Hb level, compared with TD patients. Other studies have demonstrated that transfusion dependence or transfusion intensity can negatively impact OS in patients with MDS 10, 12, 23, 24. One reason for the advantage of TI patients compared to TD patients might be that MDS is detected in an earlier stage in these patients and thus patients can be observed for a longer time.

There were fewer patients of Int‐1 IPSS risk status in the TI (Hb ≥10 g/dL) group than in the other cohorts. This is to be expected, as these patients did not have anemia (defined as Hb <10 g/dL), which impacts on the number of cytopenias and, consequently, the IPSS score. Although the difference did not reach statistical significance, TI patients with Hb <10 g/dL had shorter OS than TI patients with Hb ≥10 g/dL, suggesting that the severity of anemia in patients with TI del(5q) MDS is an important risk factor for early mortality. Thus, it may be valuable to consider active treatment options, rather than supportive care measures alone, in some TI patients, as a means of reducing the risk of disease progression and death while improving health‐related QoL. One possible option is treatment with lenalidomide.

The interventional part of this study examined the effects of lenalidomide on Hb levels and QoL in TI patients with Hb <10 g/dL who were enrolled in the phase 2 RevMDS trial. Although the sample size was small and data for some patients were incomplete, two points of interest emerged. Firstly, baseline scores for physical and fatigue QOL‐E© were correlated with baseline Hb level. This is perhaps not unexpected, but indicates that patients perceive an impact of moderate anemia on these aspects of their QoL. Secondly, treatment with lenalidomide led to early and significant improvements in Hb levels, which correlated with a significant increase in QOL‐E© physical scores. Noteworthy, in the cohort of TI patients, all 12 patients were women. Women per se have a longer OS than men and may also have different response patterns to lenalidomide. Hence, the difference between patients <10 g/dL and TD patients could be in part attributable to this bias.

These data are consistent with previous reports describing an association between QoL and both Hb levels and transfusion dependence in patients with MDS 20, 25, 26, 27. In a prospective observational study, Hb level was found to be the most important independent predictor of QoL 26. In that study, the mean baseline Hb level for TI patients was 10.9 g/dL, and 10.3 g/dL for the total study population—somewhat higher than in the present analysis. Although the prospective study found significant concordance between patients' self‐assessed QoL and physicians' assessment of patients' QoL (particularly using the QOL‐E© tool), physicians were more likely to make systematic optimistic errors, tending to rate performance status as good when the patient reported poor QoL 26.

In conclusion, there is increasing recognition of the value of patient‐reported outcomes in helping to guide treatment selections for patients with MDS 28. Although the risks of adverse events, such as severe cytopenias, and benefits of early lenalidomide treatment, in terms of morbidity and mortality, should be further explored in a randomized setting, the present analysis suggests that patients with moderate anemia and poor physical QoL perceive a need for treatment before the onset of transfusion dependence and may benefit from early treatment with lenalidomide.

## Conflict of Interest

Oliva and Balleari have received honoraria from Celgene Corporation for advisory board membership; Oliva, Lauseker, Palumbo, and Latagliata have received honoraria as speakers for Celgene Corporation; Lauseker has received consultancy fees from Celgene Corporation; Germing has received research grants from Celgene Corporation; Spiriti, Poloni, Cortelezzi, Sanpaolo, Volpe, Ricco, Ronco, Alati, D'Errigo, Santacaterina, and Kündgen have no conflicts to disclose.
